# Making sense together: dance improvisation as a framework for a collaborative interdisciplinary learning processes

**DOI:** 10.1186/s12868-024-00907-7

**Published:** 2024-10-16

**Authors:** Lisa Nelson, Julien Laroche, Nara Figueiredo, João Fiadeiro, Joseph Dumit, Asaf Bachrach

**Affiliations:** 1 Independent artist, Vermont, USA; 2 Center for Translational Neurophysiology of Speech and Communication, Italian Institute of Technology, Ferrara, Italy; 3 EuroMov Digitial Health in Motion, Univ Montpellier, Montpellier, France; 4 Department of Philosophy, Federal University of Santa Maria (UFSM), Santa Maria, Rio Grande do Sul Brazil; 5 Independent artist, Lisbon, Portugal; 6 Science and Technology Studies, University of California, Davis, CA USA; 7 Structures Formelles du Langage, UMR 7023, CNRS, Paris, France

**Keywords:** Dance, Joint improvisation, Phenomenology, Research-creation, Interpersonal dynamics, Collective sense-making, Intersubjectivity

## Abstract

This editorial outlines the outcome of an interdisciplinary session on collective sense-making through dance improvisation, which took place during the ‘Neural and Social Bases of Creative Movement’ workshop. We argue that joint improvisation practices place the scientist in a privileged position to reveal the nature of cognitive and creative behaviors.

## Prologue

Three people are moving in the space with their eyes closed for 3 min. Other people in the room are observing them and raise their hands occasionally. They report:

*The hands in the air signal that the observers - just like the practitioners - are meeting not in what makes sense but in the edge of the senses. Somewhere in-between meanings*, *directions and sensations.* (Fiadeiro)

*Some experiences fit into a projection of ourselves into the selves we are (wanna be) in relation to others.* (Figueiredo)

When I fell, *I lost all connection to my partners in the trio. This loss makes me realize the extent to which I did feel part of it just before the fall.* (Bachrach)

*We enter into the space created by where the players face*, *where they are “not.” I’m feeling (for) the rhythm arising*, *watching and listening to a pattern as it emerges*, *with skin*, *with eyes*, *with the orchestra of the senses*. (Galanter)

*Closing the eyes tricks us into experiencing the mind of and in the body. I am noticing the tuning of thinking with the mind with thinking with the body.* (Nelson)

*A flow that lifted me*, *oriented me. It was not felt as coming from the inside*, *but coming from a larger context that was nurturing this inside.A shift from self-to-world to world-to-self sense of agency.* (Laroche)

## Dance improvisation as research methodology

The International Workshop on The Neural and Social Bases of Creative Movement^1^ held in April 2022 in Fairfax, Virgínia, gathered researchers working on diverse topics such as neuroscience of movement and dance, movement physiology, dance and technology, dance therapy, and dance in education. Traditionally, workshop symposia bring together researchers to present work they have already done. However, the group we formed, composed of choreographers, dancers, philosophers, anthropologists, and scientists, worked as a team to prepare a participatory collective session. The invitation to the group members was to speculate, or imagine, what a danced workshop could be (rather than a workshop about dance), a session *of*, rather than about, collective sense-making.

This paper reflects our shared experience that improvisational dance offers research methodologies that dialog with, and can contribute to, current scientific practice. The process we put in place before and during the Workshop was an invitation to research collaboratively with collective improvisation as the ground of our experimentation and communication. We saw ourselves as an emergent “community of practices” [1] since we came from different backgrounds with a collective commitment to learn together, and from each other, in a shared practice, without a structural or intellectual hierarchy. Such a process eschews the hierarchization of disciplines (e.g. taking one discipline, such as dance, as an object of study by another, such as science). Rather it favors cross-pollination across perspectives and the emergence of renewed knowledges and refined points of view by looking at disciplines through the lens(es) of each other. The excerpts quoted above come from individual reports during phases of reflection after practice that then guided the elaboration of our participatory session during the workshop.

As a group, we are connected by intersecting interests including learning (all of us are educators in our respective fields) and improvisation. Together we explored the relevance of personal and interactional experiences for understanding the ways in which moving cognitive beings make meaning together. We came together to learn from each other’s practices and prepare a situated learning experience of collective sense-making during the conference. Given our different backgrounds, we needed to learn *how to learn together* as often our respective knowledge bases and knowledge making practices were outside the epistemic framework of the others’. Generally scientists need to learn how to learn by immersion (through practice and direct first person experience) rather than by outer observation and deduction. Dance artists often need to learn how to learn through formal modelization, and explicit hypothesis formation and testing. Our collaboration was grounded in the specific experience of each member of the group as artists, researchers and educators. We thus meshed our experiential and theoretical backgrounds based on (1) the artistic and pedagogical practices of improvisational choreographers Lisa Nelson and João Fiadeiro, who proposed practices that the seven of us explored during 5 days of preparation before the workshop; (2) the enactive theoretical framework [2, 3]; (3) the epistemological framework of feminist scholars Donna Haraway [4] and Karen Barad [5, 6].

As dance improvisation is simultaneously an observation of oneself and being observed by others, the intersubjective nature of collective improvisation allows us to notice in real time how our personal experiences are constructed from multiple perspectives. Being the subjects of our own experiments is key for our proposal, for we believe it will contribute to bridge what is called the blindspot of science [7], namely, the divide between a supposedly objective world, which is investigated by a scientist in a “god’s-eye view of nature,” and the lived world that we experience subjectively. Such a divide neglects the fact that we participate in and create the world we inhabit. For this reason, accessing and exploring our experiences is the best way to avoid the false distance imposed between the observer and the observed by so-called objective methods of investigation.

In addition to affording this particular epistemological bridge, dance improvisation has other features that make it particularly pregnant for the study of collective sense-making. Dance improvisation creates a space of non-verbal communication. It makes use of non-lexicalized body movements rather than words (or lexicalized gestures as in sign language or mime). There is no expectation for a single common meaning or narrative to emerge. As a consequence, this practice focuses the attention of the participants and observers on the *how* rather than the *what* or *why.* It invites a pre-symbolic form of semiosis where multiple voices or narratives can co-exist (Massumi’s dissensus [8]), where questions arise and are left open rather than answered [4]. Based on movement, the practice highlights the materiality of cognitive processes [9] and intersubjective dynamics (kinesthetic empathy; [10]). When observing dance, and in particular improvisation, the observer engages in a real time editing process (choosing what to look at, how to frame it, when to make a cut…) and meaning-making where they have to deduce (and/or participate in inventing) the rules of the game.

During our shared time before the workshop, we explored several ways in which we make meaning: interacting with the surroundings (space, objects, and each other) through movement and spoken language, alone and as a group; making use of scores, which are sets of constraints or rules for participation. For instance, by closing our eyes, we experience how it affects our reliance on other senses during our interactions. In enactive terms, these are processes by which meaning emerges from the processes of interaction in which we participate [3, 11]. In *situated epistemological* terms [4], this acknowledges the social construction and perspectival nature of knowledge required in any kind of objectivity. In *ontoepistemological* terms [5], this is how objectivity emerges through our intra-active relationality. During our session in the Workshop, we offered those very practices, instead of their description, creating a setting in which people could best experience our perspective on meaning-making by *doing*, as opposed to passively attending to the presentation of other-produced content. One of the practices we chose to share with the Workshop was the Blind Unison Trio (henceforth BUT), a collective improvisation Tuning Score created by Lisa Nelson as a performance score and co-teaching/learning format. BUT makes apparent and highlights both congruences and non-congruences in collective experience and ways of being/dealing with the uncertainty that ensues. In this paper, we focus on this score and more specifically on our individual experiences with it and what it taught us. As distinctive of Lisa Nelson’s Tuning Scores, BUT unites dance and the performance of observation. It draws from genetic and acquired skills of survival: how we look at things, what we “need to know,” the perceptual process of editing spontaneously in order to make meaning out of any moment. By making evident how we sense and make sense of movement from inside and outside, it exposes idiosyncrasies in our perceptions and beliefs about space, time, action, and desire, and provides tools for communication and feedback.

## The BLIND UNISON TRIO (BUT)

BUT is a performance score and co-teaching/learning format for a group of four or more people. Three are moving, and the others are watching. Directions for participation describe the unfolding of this score and how it works:.

### Direction for movers

Phase One—3 people, eyes open, enter (i.e., walk into, step into, move into, dance into) a space (i.e., a room, an environment) with the intention to come to (i.e., agree upon, tune into, negotiate, establish) a visual unison stillness (i.e., all in the same position or shape) that will be a starting place.

Phase Two—When you feel agreed, one of you calls BEGIN which signals all to close eyes and start the task to “imagine what the other 2 are doing and do the same thing” for the following 3 min. The call also signals to a watcher to start a stopwatch.

### Direction for watchers

—Notice the movement of your attention from one aspect (people, space, architecture, emotion, detail, organization, time, sound, your own body) to another. Notice what catches your interest in the activity in the whole environment (e.g., a bird passing through a window, your comfort in your body).

—Keep aware of the safety of the movers and call out OPEN (eyes) or PAUSE if you sense a mover is in danger of injuring themselves or the environment (colliding with something or somebody). The movers reclose their eyes as soon as they perceive their safety, and continue on.

—After watching a few runs, add the task of raising your hand whenever you perceive a unison (or synchronicity) of elements—e.g., shape, spatial organization, rhythm, timings (of beginnings and endings of movement or stillness), your expectation, or simply feeltouched by what you’re experiencing.

Phase Three—At 3 min, the timer/watcher calls END and movers and watchers report on their experience.

## Figuring it out

During our preparation for the workshop session, we quickly came to realize that the collective process we were engaging in for preparing the session about sense-making, was itself a form of collective meaning-making exercise. In the words of Fred Moten [12]:

*in my mind I’m just running through the whole range of possible permutations of the phrase figure it out*, *work it out…there’s this thing where you kind of figure*, *okay*, *let’s get together to see how we can figure it out*, *let’s get together to see how we can work it out*, *let’s get together to see how we can get out of this. And we’re interested in the moment at which this kind of weird inkling or transformation might begin to occur*, *in which you realize that what we’ve been trying to figure out how to get to*, *is how we are when we get together to try to figure it out*.

We tried out a variety of scores and explorations involving movement, object manipulation, speaking, and writing, and we shared our experiences. The experiential grounding of our exchanges helped us see that despite the disciplinary differences and the differences in lexical fields that come with them, we were often engaged with similar questions such as these, formulated by Nelson: What do I know about my perceived world? How do I organize myself to look at, to watch, to see? What is the beginning and end of a movement, a sensation, a thought? Can I feel my body going in and out of sync with my intention, my awareness, my desire? What thread am I following today? What is the filter of my attention?

In this section, each of us shares a short writing about our BUT experiences. This is a textual analogue to the hand-raising during the score. Like the hand-raising, it is a public expression of our personal interest. Like the hand raising, it is not about coming to agreement or consensus but highlighting the differences and trimmers (intersections: *chevêtres* in the sense of Deligny [13]) of our personal interests in the practice of this form. These differences are seen as the key and force of a community of practices approach. The last section, the epilogue, was written by Dumit, who was not able to join the in-person process during the Worskhop. Dumit followed and commented regularly on the process via Zoom. As a consequence, he became the exterior eye of the process and the ensuing article, and it is from this perspective that he wrote the epilogue.

## The improvisation of observing things

### By Lisa Nelson

I’ve negotiated a unison starting place with 2 others that has me paused, eyes closed, reaching upwards from my toes to tips of fingers. I recall another body close to mine. Reading my internal organization, what can my body do from here? I am acutely aware of the sensation of the movement of my attention, in and out of my body and thoughts. How will I survive the constraints I perceive? Which of the myriad signals from my inner and outer environment will I follow? I am tuning myself to remember the future. Will I like it there?

While watching, waiting for a suspended stance to begin to move thrills me. Though my animal senses can’t help but make predictions, my reasoned mind knows I’ve no way to know what to expect. I enter a state of unattached fascination, like watching a flock of wild turkeys in my yard. Can I memorize this attentional state as a physical organization I can recreate at any moment?

Watching BUTs reveals that my senses delight in perceiving synchronicities. First of all, the synchronicity of feeling touched by something, of having a felt perception at all. Oh, one started moving, then immediately another, then another—a canon, like a movement moved through their bodies like the wind. The room moved; or the space moved them, and me. That’s the best.

A choreographer’s textbook emerges from watching the non-visually navigating bodies in space. This accidental choreography reveals the palette of the dancer’s craft: facings, levels, rhythm, phrasing, unison, “floor” patterns, stop and go, enter and exit, body tone, affect, shape, speed, repetition, attention, intention, light, sound. Is it possible to NOT notice these elements? To NOT be entrained to our human animal perceptual wiring to note stability and change? Playing close to my animal instincts as a watcher (simultaneously predator and prey), I can *feel* my human nature in the mix. What do I like to look at? Whether or not my desire to see beauty is distinctly human, I’m looking to fall in love with the image. And this accidental choreography consistently feeds my aesthetic appetite for organically arising form.

As I watch one trio after another after another in a specific playing area and notice what intrigues or pleases or baffles me, I wonder: how are they attending to the task/the space/the time/their desire that made THAT happen? Watching and doing and watching and doing demonstrates unique possibilities that inform my next try. I notice that certain values—an aesthetic—emerge by being iterated unconsciously amongst the group, even as each individual has agency to explore their own interest, to follow their own learning curve. Reports illuminate these values, perhaps emerging as shared questions. Verbal reflections-translations of experience also articulate unique strategies to survive the score and further inform the play on both sides of the action.

## Tuning our intuition: learning to learn intersubjectively

### By Asaf Bachrach

Over the years of practicing the BUT I have been struck by what I named as “learning” that happened throughout each iterative session of the score and through the layering of my meetings with the score across time.

My questions had more or less the following form:

i. What is it that we are / I am learning, or what improves?
ii. How can learning happen despite the fact of having our eyes closed with no visual communication within the trio and no access to the feedback from the observers (hand raising)?
iii. What are the features of the BUT apparatus that bring about this learning.

The following quote from one of our group members (Laroche) describing his experience as part of the trio is revealing:

*A thing about this moment was an atmosphere of « confidence », the confidence that I was into « something » (in the sense, a something that is happening at a larger scale than me), that I was following a flow that surrounded me, rather than was inside me. A flow that lifted me, oriented me. It did not feel as coming from the inside, but coming from a larger context that was nurturing this inside.*

Laroche’s description is very close to one of the (many) ways intuition is defined:

*coming to direct knowledge or certainty without reasoning or inferring. (Merriam-Webster)*.

Intuition is often defined negatively—in opposition to reasoning or conscious inferential process. In the BUT, the three eyes-closed dancers cannot rely on habitual sources of information and habitual heuristics to achieve unison. As a consequence, the explicit, vision-based form of reasoning becomes useless; instead the dancer is invited to function intuitively. Nelson writes:

*closing the eyes liberates … what? the tyranny of mentalized choice making we fill the space between one chosen direction and another with following and unconscious patterning.*

I would argue that Laroche’s feeling of being guided by a sense of flow is a phenomenological experience of intuition. Relatedly, Järvilehto [14] proposes that “flow can be construed as intuitive action, whereas intuition can be thought of as cognition in flow”. Intuition is often described as (involving) a form of affect or feeling. Bastick (cited in [15]) defines intuition as : “feelings which guide our common actions”. Indeed, another participant of the same trio, Figueiredo, describes her experience in terms of feeling:

*I just felt like reaching in different directions*, *I felt it as a kind of searching for the others and feeling the space around me.*

I would argue that (the study of) intuition is also relevant to the spectator role in BUT. First, the spectators are invited to watch, for 3 min, 3 persons engaged in an intuitive quest. Furthermore, the spectators are asked to raise their hands when they feel unison. Nelson, when teaching the score, insists that the hand-raising is not only about visual unison but a feeling of unison or synchronicity, involving the body of the spectator as well. Once again, we are in affect territory rather than veridical opticality. In my experience of practicing BUT, I often feel as if my hand rises by itself or is ‘in sympathy’ with the ongoing dance (rather than being a gesture indicating a conscious decision). However, the standard definitions of intuition fail to capture a fundamental aspect of the way intuition plays out in the BUT: intersubjectiveness.

At the phenomenological levels, both Laroche and Figueiredo (in the quotes above) explicitly name this. Laroche talks about

*a ‘something’ that is happening at a larger scale than me…It did not feel as coming from the inside*, *but coming from a larger context.*

Figueiredo describes searching for the others. Standard internalist/cognitive/psychological accounts of intuition consider it as a purely individual phenomenon, with no place for the inter/trans-subjective. I suggest that the BUT score is a first person plural study (Extending Varela first person laboratory [16]) of intuition as a trans-subjective phenomenon.

Let us start with the observations of practitioners that being un-sighted for 3 minutes does not cut oneself from the larger (sensorial) context, but highlights sensorial information often neglected (or less attended to) when vision is available, such as sound, touch (including of changes in air movement close to the skin), smell and temperature gradient. The absence of ‘veridical’ visual form matching (as well as inter-personal channels such as mutual gaze) to coordinate with the other two, the dancer’s attention is brought to the multiplicity of feedback loops between their movements and the flow of incoming sensory information, a first person study of sensory-motor contingencies. Action and perception are felt in their inseparability [2]. Intuition, as practiced by the BUT trio is a wholly embedded and extended form of cognition where the perceiver/actor is invited to experience themselves non-dualistically as part of the ongoing event rather than as separate minds observing a separate ’world’.

However, this is not the end of the story. The description of intuition above would have been adequate if there was a single dancer, yet in the BUT there are three (and the spectators). As a consequence, the action-perception loops of one dancer mesh with those of the other two. This situation is not very different from the perceptual crossing paradigm of Charles Lenay and colleagues. Lenay [17] proposes that our own sense of subjectivity or self (as well as ‘the other’) emerges through the encounter/crossing of our own perceptual system with that of another. Lenay’s enactive account of the emergence of the self is strongly inter- or trans-subjective (see also Stern’s account of the emergence of subjectivity during development [18] and Reddy’s [19]), and so is the form of intuition or intuitive movement engaged in the BUT.

One ‘classical’ debate in the intuition literature is the veridical status of intuition (is intuition always true? ). In the context of the BUT, this issue arises concerning situations such as the account of Laroche above. Does his “confidence that I was into « something »” need to correspond to something we would call real or true? How would we even go about figuring it out? (Should it correspond to hand raising by spectators? Should it correlate with more synchronized movement or more similar body shapes? Is the alternative to a criteria of correspondence with some ‘external’ measure of objectivity an endorsement of a solipsistic stance? Though in many respects different, the same question arises regarding the hand raising by the spectator. Is there a sense in which hand raising can be wrong? Is there a procedure to verify the correctness of such a gesture? The philosopher and theoretical physicist Karen Barad writes:

*Objectivity cannot be about producing undistorted representations from afar; rather*, *objectivity is about being accountable to the specific materializations of which we are a part.* [5].

The philosopher Brian Massumi [20], building on Henri Bergson writes:

*We call instinct, in its aspect of lived intuition, the sympathy that transports us, with a gesture effecting a transformation-in-place, into the heart of a unique event that is just beginning, with which our life will now coincide, but whose outcome is as yet unknowable, and consequently inexpressible, laced as the movement toward it is with supernormal tendency.*

BUT offers an experience of a Baradian objectivity, one where “to know is to become entangled.” I suggest that intuition is entangled knowing, operating, as suggested by Bergson and Massumi, through sympathy. Learning (and teaching) of, and through, such ways of knowing is not a matter of transmission of pre-packaged information from one individual to another, but a tuning of body-minds, sharing a lived context.

## Interactive tuning: learning to be together

### By Nara Figueiredo

As a philosopher, my first question regarding improvisational dance practices, such as BUT, is about the moments of interpersonal tuning. In watching others and watching the videos in which I participate in the performance, I can see that we all notice and, many times, agree on moments of unison. However, when participating in the performance, although I was highly alert and trying to stay tuned, I didn’t experience a *feeling of being in unison*, which could, in principle, correspond to a certain objective tuned^2^ configuration^3^. When I closed my eyes, I felt more a sense of searching for others, and sometimes that they were nearby. This led me to the question of what constitutes the unison. After some thought and talking to the group and reflecting about this question, I learned that I should “stay with the trouble” [4] a little longer, allowing time, space and our activities to lead me and unfold unfinished configurations. I also learned that the very question of what constitutes the unison is the result of an expectation of uniformity, permanence, standardness and artificiality, based on our tendencies of objectification. Despite learning this, the philosopher in me doesn’t rest. My desire for answers is still vivid, and I have been searching for other ways of understanding those interactions. Here, I try to avoid standardized conceptual definitions of constitution by relating my experience with the improvisation score of BUT to other ideas where I find kinship. *Active meditation* [21] is one of them. *Letting be* [22, 23] is another. In this section, I present these ideas highlighting the learning processes that are involved in them and finish by suggesting that both interaction and conceptual clarity involve a kind of convergence.

Thich Nhat Hahn [21], a renowned zen master, talks about active meditation first as something done in times of need. He gives the example of going out in meditative action to care for people that had just been hit by the bombs during the Vietnam war. That is a very intense moment and he is calling for meditation to be something done jointly in support of society, as a practice of connecting people. He proposed *meditation in action* as a social interactive practice to promote collective well being, connectedness and peace. In practicing the tuning games I can see its close relation with the concept of meditation in action, not in virtue of the need to help others in times of intense suffering, as in the example mentioned above, but in virtue of the experiences of connecting with others in ways that preclude overthinking and inner narratives. Like meditation in action, the interactive tuning games, such as BUT, can also be seen as a practice of care, but one that addresses a different kind of trouble. Namely, our current collective existential crisis, as connected (and sometimes disconnected) beings on Earth - as pictured by Haraway [24].

*Letting be* is a way of inhabiting the world in which we acknowledge our fundamental condition of being interconnected to others. This means that we are co-implicated in our relationships: we are always in part determined by the ways in which others act upon us and are also determining in part the beings of others by the ways we act upon them. This is how people’s actions position participants in relationships and therefore form inherent and natural tensions between how people see themselves and how they are taken to be in interaction. The paradox of letting be is that “other’s actions *never* simply ‘let us be’ or leave us free to be who we are. Other’s actions *always* situate us and determine us” [22, p. 196] and, mutually, our actions never just let others be. Because of this inherent tension in how we determine ourselves and others and are determined by others, there is a constant need to learn a kind of balancing in our relationships, a way of interacting that neither over-imposes our own normativities or ways of being nor under-imposes them [3]. We have to learn to let others be and also act in ways that promote others to let us be.

*Interactive Tuning*^4^ practices provide safe opportunities to learn that and, consequently, to learn to be together. It is a process that allows for new ways of understanding ourselves, our surroundings and others. When it is facilitated by group improvisation cues, we pay attention to many signals that are part of feeling the other and oneself — noticing small gestures, like change of gaze, body tension, speed and hesitation in gesturing or moving or speech, and how it relates to standard habits, whether it is common or uncommon, accidental or intentional, or if it is a general ability or inability and so forth. Take, for example, the moment of BEGIN in BUT, described above. It is a moment when people notice an agreement in their tuning, and one of them makes the call. In this visually achieved unison, my experience was of searching for balance in the situation, an attempt to equilibrate my own normativities^5^ with the others. This search, I believe, involves a kind of *readiness to co-create togetherness*. And the agreement involves a kind of convergence in acting, or harmony.

If we think about our daily activities in our social lives, the co-creation of meaning can happen in many different ways. Consider, for example, an act of mis-pouring wine into a crystal glass. It can be just a glumpy miscalculation of the space. Or it can be a general handling inability. Or it can be a reflection of a state of anxiety, among other things. Noticing what is happening involves very fine and quick perceptions, from the second and third person perspectives. Noticing it from the first-person perspective involves a deep self-knowing. Even if one (first, second or third person) cannot determine in which case the wine pouring action falls into, one is still *able to act upon it*, leaving open the chances of each possible interpretation and actually contributing to construct one. It can also be the case that a new interpretation superimposes on a previous one and transforms it^6^. And this is one of the ways actions acquire meaning. The main difference I want to highlight between our daily activities and interactive tuning practices is, in Nelson’s words, that ‘nothing is at stake in the tuning games’ - As opposed to daily life, in which our actions and other people’s actions situate ourselves and weigh in on the construction of our social selves. This makes it a safer place for us to learn to create togetherness^7^.

I believe that this open fine perception, understanding, and collaborative construction by means of subsequent and overlapping actions and interpretations involves a suspension of judgment, a holding up (or blinding) of expectations, a preclusion of inner narratives and overthinking, of pre-given societal practices, interpretations and impositions, an openness to connect with the other. It also involves a kind of generosity or kindness in constructing the sequence of actions and meanings in an inclusive way, not pushing others to a position of alienation of the co-participative action nor downplaying or overplaying one’s own normativity. This, in my view, is what Maclaren [22] and De Jaegher [23] call *letting be*. It is a kind of attitude that involves embracing uncertainty, ambiguity and contradictions, and letting ourselves and others *keep going* in spite of them.

There is yet more to be learned from these experiences. It refers to the very process one engages in when writing about them, the process of conceptualizing them. Bringing forth an understanding from the experiential level to the conceptual one is a complex activity, involving evolutionary and socially developed languaging abilities (see [25] for a short explanation) of abstraction, synthesis and objectification. It involves also an ability of *provoking* (or eliciting) *with words*. This *wordly provoking*, let us say, must be done while minding the need to avoid undesired ambiguities and contradictions. These are often caused by the very use of language, which is a highly regulative practice, but they are also present in our very ways of experientially knowing. In describing this process of maturing conceptual understandings of experience, I can identify three levels, dimensions, or moments of sense-making^8^: (i) the intersubjective connection, (ii) the ability to bring forth words (and symbols in general) based on our repertoire of verbal practices, and (iii) the ability to actively construct verbal meaning with them, which, I believe, involves a back and forth mechanism of checking whether chosen words play a role that engages with (constructs with) these experiences^9^. This checking stage is better done interactively, but, due to our training and ability to enact inner dialogues, it can also be performed individually.

Despite the steps involved in this process, I believe that it is fundamental that we take it as a relational process. A relation that involves not only people, experiences and words (or symbols in general), but also what and how words provoke. When I imagine a state of conceptual understanding of what constitutes the unison that may be fostered by time, space and activity and I think about the very connectedness I feel and practice when improvising, I can see both as moments of a kind of convergence. I want to think about this convergence in Barad’s terms [5]. She talks about how lightning is a phenomenon of indeterminacy happening in a relation between the clouds and the earth and how there seems to be (or is) a connection that has no specific sender nor recipient up to the point in which it occurs. In the kind of conceptual understanding that I’m referring to, there may be several layers of convergence, involving people, experiences, words, history, time, perspectives and also what and how words provoke. This is the co-creation of verbal meaning.

## Exploring togetherness and the interplay of subjective and objective states in collective action

### By Julien Laroche

Acting cooperatively with others provokes an intrinsic motivation to learn [26] and generates interaction dynamics that favor or even accelerate the learning of novel behaviors [27]. The experience of acting jointly thus seems to bring about peculiar neural and behavioral dynamics that can enrich our capabilities [28], and studying behavior in interactive contexts should help us better understand the nature of these dynamics. A strong experiential correlate of acting together as a group is the sense of « togetherness » that can emerge from our interaction: the impression of temporarily forming a strong bond, and the feeling that agency originates in a collective entity, rather than in a collection of individual movers. Dancing with others is particularly prone to generate such a feeling [29]. Finding the causes that elicit such states and the consequences they promote, as well as establishing correlation with other aspects of behaviors and experiences, is a current subject of investigation in cognitive sciences, and collective dance improvisation is a privileged window on this phenomenon.

At the center of this issue, as often with psychological inquiries, is the tricky issue of the relation between the subjective and the objective states in which persons find themselves to be - in other words, between the quality of experiences, and the patterns that we can uncover and quantify in the material world. In the context of collective action, to what extent do feelings of togetherness overlap with the temporal coordination of behaviors ? It is often thought that these two aspects go hand-in-hand [30–32], but sometimes the coordination between the dancers’ movement coordination and the feelings of togetherness as judged by an audience don’t match [33]. Furthermore, even when there is a match, it is still unclear whether feelings of togetherness are a scaffold to collective actions or merely an outcome of the coordination of behavior across persons.

Similarly, one can wonder if it is the state of the coordination itself that matters, or the way it is subjectively perceived by interacting participants. This opens questions about the respective role of reflective and pre-reflective processes: is togetherness explicitly perceived, implicitly felt, or an interplay between both ? Moreover, a strictly linear relation falls short of explaining why imperfect synchronization between performers carries an aesthetical value [34, 35] and provides a context to deepen this feeling [27, 36]. In the latter case, a possible explanation is that togetherness bears on interactivity (the degree to which individual behaviors become contingent on each other), rather than the precise coordination itself.

The BUT brings disruption in these traditional angles from which we question the phenomenon of felt togetherness. If this feeling bears on the coordination of action across persons, what happens when we lose sight of our partners and the physical results of our interaction (or lack thereof)? During the practice, I felt clear shifts in my lived experience regarding togetherness: there were abrupt transitions between moments where I was reflectively questioning my ability to dance with others in such conditions, and moments where I had an intuitive confidence that my movement was actually part of a group flow. In the former case, I felt caught in a self-to-world logic of adaptation where I tried to fit my movement to the environment as I explicitly imagined it. In the latter case, I felt a world-to-self sense of causality where the environment as I experienced it implicitly seemed to direct my movement. With these qualitative shifts in mind, I cannot rely anymore on previous explanatory schemes.

Given the strength of those shifts, could they really be a mere projection or the product of a random imagination ? If not, on what grounds or aspects of reality (physical or psychological, for what it matters) can these shifts happen ? Did I feel togetherness with others because I sense my responsibility of my own action is de-centered and find causes in others, simultaneously as I feel having some agency over their own behavior ? Moreover, during my practice, the appearances of these shifts were unexpected, surprising, yet they seemed to have found counterparts in observers’ judgment of synchrony/unison. What factors caused them to perceive unison at some points more than others ? Were the feelings of togetherness experienced respectively from first and third person perspectives synchronous themselves ? And did the fact that observers were looking at us (and had to express their feelings of unison) change how I conceived and sensed togetherness myself ?

Experiments using simple movement interaction do show a relation between feelings of togetherness and some fluidity, smoothness, and sense of confidence in movement dynamics [32]. But is it my sense of confidence in the interactive activity that smoothes my movement and rubs out my hesitations ? Or is my movement fluidly guided by the collectiveness of our motion when we are more in sync? This question struck me as I was practicing the BUT, as I felt more comfortable with my own movement when I was confidently feeling that I was participating in a larger ensemble of movers. On the contrary, when I was in a phase wondering about the coordination of my movement with those of my partners, uncertainty raised and confidence dropped. It made me realize that there was a strong correlation between reflectivity and confidence: uncertainty about my attunement to others was brought to attention during reflective phases, and confidence accompanied a rather pre-reflective feeling about my participation in a collective motion. Was the sense of confidence a prerequisite to fully participating in the attempt of moving collectively ? Was it necessary to get a sense of joint action where the reflective self dissolves and collective dynamics seem to inform my movement despite the absence of cross-feedback ? Or were aspects of such collective dynamics the source of my sense of confidence ? Could there be a mutually reinforcing relationship between those two aspects ?

In effect, after I sensed a profound confidence in the togetherness of our movement, I noticed the sounds we were producing on the floor were forming some sort of acoustic collective « choreography ». I wonder if those sounds were pre-reflectively guiding my actions and my sense of togetherness, or if the feeling of togetherness made me notice them in the first place ? More generally, it made me realize that when vision, on which we tend to rely almost exclusively when assessing the togetherness of a group of movers, is “out of the picture”, we might sense this togetherness through the cues provided by other modalities.

In sum, the practice of the BUT puzzles some of the conceptual mappings that structure my own thoughts and work and those of my field of research.Thus, the potential of such practice or installation does not reside in successfully answering previous questions, but in posing new, relevant ones. It can help me gauge my own concerns and reveal the caveats of the frameworks or worldviews I endorse implicitly or explicitly. My personal experience of the practice of the BUT opens a wide array of new questions, curiosities and potential paradigms that I would like to further explore with my own practice: empirical science. More generally, through the experiences associated with collective improvisation practices, we can inform ourselves about the processes and the habitual tendencies that we endorse during our interaction. This could help to enhance metacognitive abilities and, therefore, foster self-efficacy [37] during collaborative learning, in addition to motivating us to engage in such collaborative activities where we can learn from each other through the very process of our interaction [26].

## Not to feel alone (in the unknown): a personal take on Lisa Nelson’s blind unison trio practice

### By João Fiadeiro

#### Blind

“Change blindness” is the condition we all share when we fail to notice something we don’t expect to see. If expectation blinds us, how can we blind expectation? That is the research question I experience when practicing blind unison trio. By closing my eyes when trying to preserve the unison, I can stop (or at least reduce) the impact of expectation in my decisions. Not expecting (or projecting, or judging, or taking for granted) with the eyes open is a practice reserved for few. Closing the eyes is a simple and accessible way to experience (and through experience, study) the sensation of being blind to expectation. And through that portal meet the other on the other side of the known. On the other side of knowledge. Using this strategy it’s possible to unlock multiple pathways for the imagination and the imaginary to flourish. This is only possible if we don’t know where we are. Only possible if we are lost. Here, being lost functions as an effective antidote to expectation and allows us to tune in with others in a place further (deeper) than the surface. if I’m not lost I’m not really tuning. I’m syncing.

#### Unison

Synchronization is a useful tool (which allows me to write these words and press “send” once the text is ready) but it also produces an artificial sense of togetherness. Being together out of sync is only possible if we manipulate time. And even if the word “manipulation” comes from “hand” (*manus*) - suggesting a more (hand)crafted way of existence - synchronization produces copies, not repetition. And repetition, as we know, is a necessary ingredient in order for difference - the stuff relations are made of - to emerge. Once lost, I can start falling. While falling I understand how vulnerable I am, and this is a crucial part of the experience. In order to allow the fall (into the unknown) to continue, in this moment (when I face my own vulnerability), is to resist the tendency (and temptation) to protect myself, which the body is designed to do regardless of my (good) intentions. In a way the work is to protect myself from what I want. Falling into the unknown is “contra intuitive” for any adult but it’s not impossible. It’s like falling in love (for a person, an idea, an image). In this situation, love (aka trust) is all one has to hold on in order not to collapse. Avoiding collapse is a necessary condition for the encounter to occur on the edge (where the magic happens). Not collapsing doesn’t mean to stay straight or to lie down (forms of disengagement and surrender). It means that we need to find (experience, study) an oblique quality of existence. Not vertical or horizontal, but diagonal. Not stable or unstable, but meta-stable. Not dependent or independent, but autonomous. In other words, inter-connected. Delicately interconnected.

#### Trio

Why three? Three persons, three minutes… The simple answer would be because three is more than a couple and less than a crowd. For me this would be sufficient as a justification but a more elaborated answer could be because the relation that is being studied here is not between one and two, or two and three, but between the in-betweenness of one and two, and the in-betweenness of two and three. What matters here is not the relation between positions but the relation between relations which, once in motion, complexifies and multiplies the possibilities of (dis)encounters, generating a form of manifestation that resembles the behavior of an organism. An “organism”, unlike an “organization”, drives from the ability of having mutant properties and preamble frontiers, essential qualities - in cells, people and societies - to fully experience this thing we call empathy. Between practitioners but also between practitioners and observers. In this practice observers raise their hands every time they sense that some form of tuning is taking place in what they see. The hands in the air signal that the observers - just like the practitioners - are meeting not in what makes sense but in the common senses that are in-between meanings, directions and sensations. No one knows exactly where this place is but everyone knows that they don’t know where this place is, together. And this is all is needed not to feel alone in the unknown.

How “not to feel alone”. What else is there to research?

## Epilogue

### By Joseph Dumit

“But what do you really care about?” This question arises inside of the work that we all do, as scientists, as artists, as humans. As an anthropologist of how science changes, and as an anthropologist of how scientists change (into the scientists they are from the students they once were), I am intrigued by the way that the BUT score provides us with a set of answers to these questions, in the here and the now. Not permanent answers, but answers that are typically too hard to focus on. Watching this thoroughly interdisciplinary group (improvisational choreographer & mover, cognitive philosopher, cognitive scientist & musician, performer theorist, neurolinguist dancer bodyworker, etc.) meet each other at the level of their ongoing experience and experiments is a gift.

What does it mean to learn? Is it the same as changing? If we engage in an experiment that teaches us a new mode of perception, or sensation, or understanding, are we the same person (the same thing changing)? Or are we new, someone different because our sensorium is different (even if we qualify it with “not that different”)? Hidden inside of learning research is often this contradiction between learning to be better and learning to be different. The first is measurable, and the second a bit disturbing. But of course, in the process of becoming a scientist or an artist, we don’t just become better at definable tasks, we gain new qualities, we become different and new. If we pause and contemplate this situation, we find ourselves noticing how we are new students, and new scientists/artists all the time (sometimes marked, sometimes unremarked).

In this paper, these researchers have taken up the challenge of rigorously experimenting with reflexivity, togetherness, improvisation, and attunement. These are concepts that have great intuitive sense and yet are deeply recalcitrant to metrics. Or rather, metrics can be assigned under their names but usually turn out to be frustratingly boring. Because we really care about reflection, togetherness, improvisation, and attunement. So much so that we would rather leave it out of our experiments than force it to fit their constraints. And in these moments we feel the challenge of the methods we have made our professional peace with.

But this was a conference on science and dance, so these researchers took up the challenge of researching these practices by practicing them, together. Living together for ten days, sharing their working definitions and their practices for working with them. In the end, as this paper makes clear, practicing together reflexively was the only method. Practice is a funny word in experimental science, because when you practice something, you get better at it, often you change. So even in learning experiments, there is a tendency to seek out naive subjects who do not know how to do something and measure how teachable they are (as an effect of their capacities or the experimenter’s protocol). But we (presumed readers of this text) are in the middle. We are messy. We have too many words for our experience, and yet when we are challenged to attune to each other, our words turn out to be quite inadequate. Or perhaps attunement is not something that makes sense to represent in words that won’t themselves be affected by the practices of their users.

Dancers, performers, artists have a phrase for this: Practice as Research. As this group demonstrates, you set up some conditions, a score like “Blind Unison Trio” for 5 or more participants, and you do it. The doing includes not just the three who move with their eyes mostly closed, attending to their attention, and the two or more observers who are also participants entangled in noticing varieties of togetherness. The doing also includes the reflection, discussion, diary entries, and laughter. And it includes doing it again. And again. Because in that doing again, practice reveals itself to be the research. New sensitivities are noticed, vocabularies change, and sometimes amazing forms of attunement arise that challenge preconceptions.

And something else: new questions arise that confuse what seemed to be the foreground and background. The reports reveal that someone attuned initially to bodily attention starts to ask about values; practicing attunement raises questions of confidence in another; one starts to question whether togetherness is related to meditation; another practitioner starts to ask “how not to feel alone”; they all start to realize the extent to which this particular practice folds them into a space where their stance on objectivity and subjectivity is in flux. These are all things that can be practiced because they are the results of practice.

As an anthropologist who is attentive to the (not-so-)subtle psychological effects of paradigms—something that Thomas Kuhn was deeply concerned with, as was his inspiration Ludwig Fleck, and one of their touchstones Immanuel Kant—I love the way that the BUT score unsettles the terms of each practitioner within their own thought styles. I see how this process is better described by contemporary interdependence scholars Karen Barad [6], Erin Manning [38], and Kriti Sharma [39]. There is here a shared practice of “wonder” that emerges, not as an emotion, but as a practice of curiosity, of noticing, that helps one attend to the entanglement of self and togetherness that is part of all of our collective scientific and artistic lives.

Reflecting on this, I wonder at the strategies we all have to keep these forms of curiosity at bay, off stage, when we practice our methods. So that our subject-object distinctions can maintain their integrity long enough for us to publish. There is a beautiful ethnography by anthropologist Kathryn Linn Geurts [40], *Culture and the Senses*, that arises from her fieldwork with the Anlo-Ewe, and asks how they consider a sixth sense—something that overlaps with what we call “balance”—and how it is integrated into their full moral sensorium, akin to the way that my schooling in the US assumed hearing and sight to be unproblematic metaphors for knowledge and authority. As I learned different forms of disorienting improvisational dance, Geurts’ argument continually interrupted my learning with questions of what sensorium I was developing. Reflecting on the BUT practice as research, I wonder too, whether togetherness is a sense.

What would happen if we practiced noticing and talking about when and how we feel alone and not in our professional settings? When do you feel in sync with your colleagues? When do you feel togetherness? What is your relationship to the feeling, and does it take you toward some collaborations and conferences, and away from others? What would it be like to introduce practices of attunement and reflection in these different settings, and approaching them as practice as research? Is this something you really care about?

## Data Availability

No datasets were generated or analysed during the current study.

## Footnotes

1 https://yourbrainanddance.egr.uh.edu/about.

2 I’m taking ‘tuned’ in a very general sense here, meaning a kind of connection between participants, individually identified by a feeling of being together in a similar mindset or state. This feeling also usually accompanies situations in which we acknowledge and agree with each other’s point of view, and say things like: ‘Oh, yes, I totally see that!’ It also usually accompanies situations when two people are standing in line and by just looking at each other they acknowledge that it is taking too long and it is annoying. In improvisation practices the feeling is not usually accompanied by a third acknowledged element they agree upon. It is mostly mutual acknowledgement and connection. This is easily identified by observers as objective when they see two people expressing and acknowledging annoyment to each other in a line. Or when they see two people having a great time in a conversation. It is also easily observed when two people are in love, or when they simply get each other. Although during the tuning games those tunings have always been positive for me, I take that the concept doesn’t imply they must be positive, they can also be people getting angry at each other or at something for example. This ‘connects’ them into a tuned state. I take ‘tuned’ to be a state of connection while ‘tuning’ is a process, as well put by Nelson: a movement bringing two things into a sensible relationship to each other.

3 Probably because this kind of feeling highly depends on the visual abilities of sighted people, which were not being used during the practice.

4 I will use the expression *Interactive Tuning* in order to emphasize the *connective* aspect of our interactions.

5 ‘Normativity’ can be taken in a very flexible way here, as what is happening with you, your attention, your desires, needs, whatever drives you. It amounts to the concept of living organisms in the enactive theory: “vital norms specify the conditions that guide adaptive regulation (…). Norms may also be externally imposed on the system” (Di Paolo, Cuffari, De Jaegher, 2018, p. 331).

6 If one is taken to be in a state of anxiety, and is actually a clumsy person, she can make an effort to offer this other placement of herself, by saying “Oh, I always do that, don’t worry!” in an effort of lightening the atmosphere of an encounter, for example. This is a way of projecting and insisting on a view of oneself into that relationship. She can also accept the proposed view that she is anxious and take it as an opportunity of self reflection and further develop her existential place by questioning the sources of this state, learning how to manage it and learning how to situate herself in a different place. Much of this learning, according to Maclaren (2002) is done by means of inhabiting others’ actions over here. This means that when I watch someone pouring the wine I can realize how my pouring the wine is clumsy, readjust myself and my bodily actions and re-situate myself as a skilful wine pouring person. This is done both by how I can see myself based on others and by means of training.

7 Although it is quite reasonable to assume that if someone is mostly over-normative (trying to impose their view and expectations) in the Interactive Tuning Game, this will eventually lead to others’ disappointment and perhaps even avoidance. But it is also fair to say that it is a safe space if compared to our daily life, in which our social situation is constantly at stake.

8 Technical concept in the Enactivist literature. It refers to the organism’s ability to regulate its actions in its surroundings in virtue of the possible implications of those actions to its own being. It posits that things/actions are essentially meaningful to organisms, when they are part of its environment and have implications for it. Here, I use it in order to elicit a general presupposition of the Enactive theory, namely, that, as cognitive beings, we are constantly making meaning.

9 These levels may well become a hypothesis of how our mental processes work, but I will take them here as a way of unpacking the very process I am engaged in now.

## Abbreviations

BUT: Blind Unison Trio
